# Microstructure and Mechanical Properties of EK30 Alloy Synergistically Reinforced by Ag Alloying and Hot Extrusion for Aerospace Applications

**DOI:** 10.3390/ma15238613

**Published:** 2022-12-02

**Authors:** Daohe Zhang, Sicong Zhao, Hongtao Chen, Yicheng Feng, Erjun Guo, Jingfang Li

**Affiliations:** 1 Key Laboratory of Advanced Manufacturing and Intelligent Technology (MOE), School of Material Science and Chemical Engineering, Harbin University of Science and Technology, Harbin 150000, China; 2 Key Laboratory of Functional Inorganic Material Chemistry (MOE), School of Chemistry and Materials Science, Heilongjiang University, Harbin 150080, China

**Keywords:** Mg-RE-Ag alloy, IEECAP, microstructure, mechanical properties

## Abstract

Enhancing the mechanical properties of magnesium alloys to meet the urgent need for their lightweight applications in the aerospace field has always been a great challenge. Herein, the effect of Ag on the microstructure and tensile properties of the Mg−2.5Nd−1.0Sm−0.4Zn−0.1Ca−0.5Zr (EK30) alloy prepared by integrated extrusion and equal-channel angular pressing is studied. The microstructure of as-extruded alloys consists of *α*-Mg grains and the *β* phase. The addition of Ag increases the *β*-phase content. The *β* phase can promote dynamic recrystallization by inducing a particle-stimulated nucleation mechanism and inhibiting grain growth, which leads to grain refinement and texture weakening. At 250 °C, the ultimate tensile strength of the EK30–2.0Ag alloy (225.9 MPa) increased by 13.8% compared to the Ag-free alloy (198.4 MPa). When the tensile temperature increased from 25 °C to 250 °C, the ultimate tensile strength of the EK30–2.0Ag alloy decreased by 14.3%, from 263.7 MPa to 225.9 MPa. Notably, the addition of Ag slightly reduced the elongation of the alloy at 250 °C; the elongations of the EK30–2.0Ag alloy and the EK30 alloy are 41.5% and 37.0%, respectively. The elongation of the EK30–2.0Ag alloy increased from 22.7% at 25 °C to 52.7% at 275 °C. All alloy tensile fractures exhibited typical plastic fracture characteristics. This study provides an effective way to enhance the high-temperature mechanical properties of magnesium alloys by Ag alloying and a special severe plastic deformation method.

## 1. Introduction

Magnesium (Mg) alloys, being the lightest metal structural material, have distinct benefits over cast iron, steel, and aluminum alloys in terms of their specific strength, damping characteristics, and electromagnetic shielding [1]. High-performance Mg alloys have become one of the preferred choices for aerospace materials [2]. The maximum flight range, load capacity, and maneuverability of an aircraft can be continuously increased with the expanded use of various Mg alloys [2,3]; however, their applications are limited by their relatively low strength and ductility [4,5,6]. To effectively address these shortcomings, adding rare earth (RE) elements to Mg alloys is a promising approach [7,8]. Multiple series of Mg-RE alloys have been successfully developed and are widely used in the equipment manufacturing field. Notably, Mg-Gd and Mg-Y, as representatives of Mg-heavy RE alloys, can achieve high strengths via the addition of large amounts of heavy RE elements; however, this significantly increases the density of the Mg alloys and weakens the advantage of their light weights [9]. Therefore, the types, ratios, and amount of RE elements added to Mg alloys should be selected appropriately to ensure that their mechanical properties and specific gravity meet the requirements of aerospace components. A good combination of low density and excellent mechanical properties can be achieved by adding a small amount of light RE elements to Mg alloys [10,11]. The Mg-Nd alloy and the Mg-Sm alloy are typical high-performance Mg-light RE alloys [12,13]. Sm has been considered an ideal strengthening element for Mg alloys due to its lower price [14]. The mechanical properties of Mg alloys can be more effectively enhanced by simultaneously adding Nd and Sm [15]. Nevertheless, the mechanical properties of Mg-Nd-Sm are still not sufficient for the new aero-engine components. Further enhancement of the mechanical properties of Mg-RE alloys can also be realized by adding Ag [16,17,18]. Zhang et al. [19] investigated the microstructural and mechanical properties of extruded Mg−10.5Gd−5.0Y-xAg−0.5Zr (x = 0.5 and 1.0 wt.%) alloys. The addition of Ag can promote the formation of the *β* phase. The *β* phase refines the grain size and causes a significant increase in yield strength. Furthermore, the coupling effect of the increased *β*-phase content and the reduced grain size results in a slight decrease in the elongation of the Ag-containing alloy. Movahedi et al. [20] also revealed that adding Ag to the Mg−8.5Gd−2.5Y−0.5Zr alloy resulted in a decrease in grain size, an increase in the volume fraction of the second phase, and the formation of a novel intermetallic phase (Mg_16_ Gd_2_ YAg). This resulted in the Ag-containing alloy having superior mechanical properties over the Ag-free alloy in the temperature range of 25–300 °C. Additionally, the incorporation of multiple elements, including Zn, Zr, and Ca, into the Mg-RE alloy is also an effective strategy for further improving the mechanical properties [21,22,23]. Therefore, the heat-resistant EK30 alloy was designed in this work.

To date, it has been difficult to achieve a leap in mechanical properties through alloying strategies, especially for high ductility. The equal-channel angular pressing (ECAP) as a severe plastic deformation (SPD) technique can prepare Mg-RE alloys with superior strength and high ductility [24]. Nevertheless, the complex hot extrusion process and low production efficiency of the ECAP are not conducive to its practical applications. In this study, to simplify the ECAP technique and improve the extrusion efficiency, the two processes of ECAP and conventional extrusion were optimized into one process named integrated extrusion and equal-channel angular pressing (IEECAP) [25]. Notably, the most outstanding advantage of the IEECAP technology over other SPD methods is that the alloy material achieves high strength while having higher elongation [25]. Although some milestones in the research related to the mechanism of the influence of Ag on the microstructure of Mg-RE alloys have been achieved, the effect of Ag on the mechanical properties of SPD-processed Mg-light RE alloys has not received enough attention and study.

Herein, the microstructure and mechanical properties of the EK30 alloy were reinforced synergistically by Ag alloying and hot IEECAP extrusion for aerospace applications. The effect of the Ag addition on the microstructure and high-temperature mechanical properties of the IEECAPed EK30 alloy was studied in detail to provide an experimental basis for expanding the application of Mg alloys in the aerospace field.

## 2. Materials and Methods

**Figure 1:**
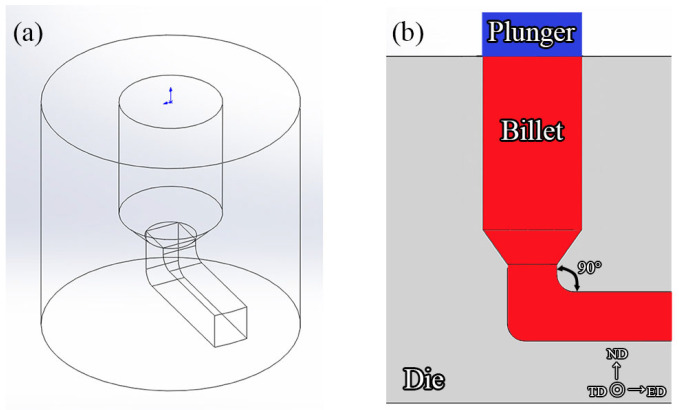
Schematic illustrations of the IEECAP die. (**a**) iso-view; (**b**) side-view.

High-purity Mg, Ag, and Zn as well as Mg−30Nd, Mg−20Sm, Mg−30Ca, and Mg−30Zr master alloys were used to prepare the Mg−2.5Nd−1.0Sm−0.4Zn−0.1Ca−0.5Zr-xAg (wt.%, x = 0 to 2.0 in 0.5 increments) alloys, which were named EK30-xAg alloys. To reduce the burn-off rate and segregation of the alloying elements, these alloy materials were melted at 780 °C, stirred for 300 s, and then cast at approximately 720 °C into a metal mold. The as-cast ingots were cut into billets with dimensions of 30 mm × 30 mm × 100 mm and homogenized at 520 °C for 8 h, followed by quenching in hot water at 80 °C. The homogenized billets and the IEECAP die were pre-heated for 1 h at 400 °C. Afterward, the billets were extruded at 400 °C into bars with a sectional dimension of 10 mm × 10 mm. The angle between the equal channels is 90° to obtain a high strain level. The plunger speed was approximately 3 mm/s. All extruded bars were quenched in water at room temperature immediately after extrusion. Figure 1 shows the schematics of the IEECAP die. The abbreviations ED, ND, and TD represent extrusion, normal, and transverse directions, respectively.

The microstructure was observed by optical microscopy (OM, OLYMPUS GX71, Olympus Co., Tokyo, Japan), scanning electron microscopy (SEM, Thermo Scientific Apreo C, Thermo Fisher Scientific Inc., Hillsboro, Oregon, USA) operating at 20 kV, and energy dispersive spectroscopy (EDS, Oxford Instruments, London, UK). The OM and SEM samples were cut along the TD-ND plane and then polished and etched with a solution of 2.1 g picric acid, 5 mL acetic acid, 40 mL ethanol, and 5 mL water. The average grain size of the alloy was determined by the linear intercept method. The grain sizes were determined based on the average of the measurements from at least five OM images to ensure the accuracy of the grain size. The texture was analyzed using electron backscatter diffraction (EBSD, Oxford Instruments, London, UK). The EBSD data were resolved using the Channel 5 software (Oxford Instruments, London, UK). The EBSD specimens were cut along the TD-ND plane, ground by 5000 SiC paper, and then polished using a SiO_2_ (0.05 µm) suspension for about 60 min. The as-extruded bars were cut into tensile specimens with a sectional dimension of 2 × 2 mm^2^ and a 6 mm gauge length. The tensile direction was parallel to the ED. Tensile properties were tested using MTS E44.304 equipment (MTS Systems Co., Eden Prairie, Minneapolis, MN, USA) with a tensile rate of 1 mm/min at 25 °C (room temperature), 200 °C, 225 °C, 250 °C, and 275 °C, respectively. The results of the tensile properties were reported as the average of no less than three repeated tests to improve the accuracy.

## 3. Results and Discussion

### 3.1. Microstructure

**Figure 2:**
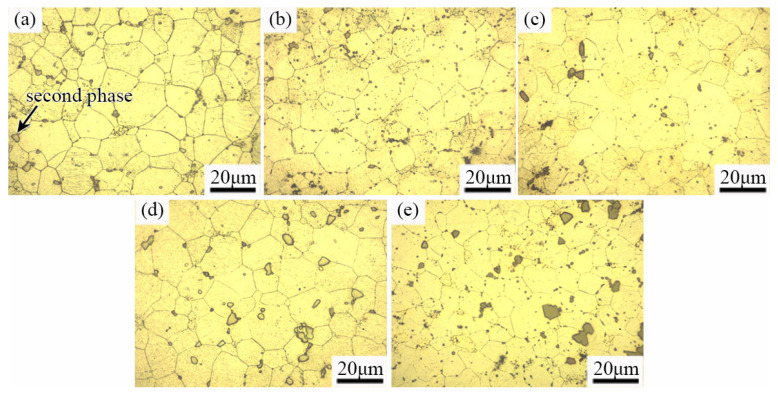
OM images of the as–extruded EK30–xAg alloys: (**a**) EK30; (**b**) EK30–0.5Ag; (**c**) EK30–1.0Ag; (**d**) EK30–1.5Ag; (**e**) EK30–2.0Ag.

**Figure 3:**
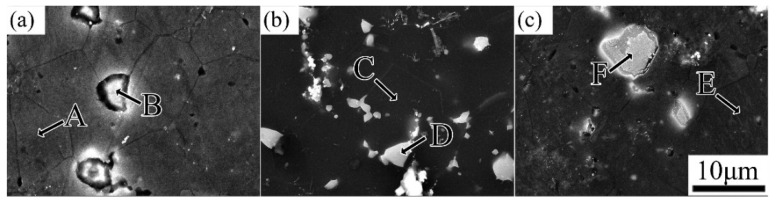
SEM images and EDS positions of the as-extruded EK30–xAg alloys: (**a**) EK30; (**b**) EK30–1.0 Ag; (**c**) EK30–2.0Ag; A-F are the locations where EDS were detected.

The microstructure of the as-extruded EK30-xAg alloys is shown in Figure 2. The as-extruded alloys consisted of *α*-Mg grains and second phase. The *α*-Mg matrix shows a fully recrystallized microstructure with relatively equiaxed grains. With the increasing Ag content, the amount of the second phase increased, and the grain size of the alloys decreased gradually. The average grain sizes were approximately 10.3 ± 0.9 μm, 8.6 ± 0.8 μm, 8.3 ± 0.7 μm, 7.9 ± 0.9 μm, and 6.9 ± 0.5 μm when the Ag addition increased from 0 wt.% to 2.0 wt.% in 0.5 wt.% increments, respectively. Figure 3 shows the SEM images of the as-extruded EK30-xAg alloy. The presence of numerous second-phase particles can be observed both at the *α*-Mg grain boundaries and within the grains, and these particles range in size from 0.5 μm to 7.5 μm. The EDS results of the *α*-Mg matrix and the second phase in Figure 3 are given in Table 1. Trace amounts of the Nd, Sm, Zn, and Ca elements can be detected within the *α*-Mg grains. The Ag content in the matrix increases significantly with the Ag addition. Evidently, most of the alloying elements are segregated in the second phase. Combining the EDS results with a previous study [26], the second phase can be identified as the *β-*(MgAg)_12_(NdSmZn) phase, and the *β* phase has good thermal stability [26]. During the hot extrusion process of Mg alloys, the second-phase particles, ranging in size from 1.0 μm to 10.0 μm, can promote dynamic recrystallization by inducing a particle-stimulated nucleation (PSN) mechanism and thus achieving a good grain refinement effect [27,28]. Most of the *β*-phase particles in our alloy systems meet this condition. Moreover, thermally stable *β*-phase particles may also inhibit grain growth during hot extrusion. Consequently, the grain size gradually decreased with the Ag addition.

### 3.2. Texture

**Figure 4:**
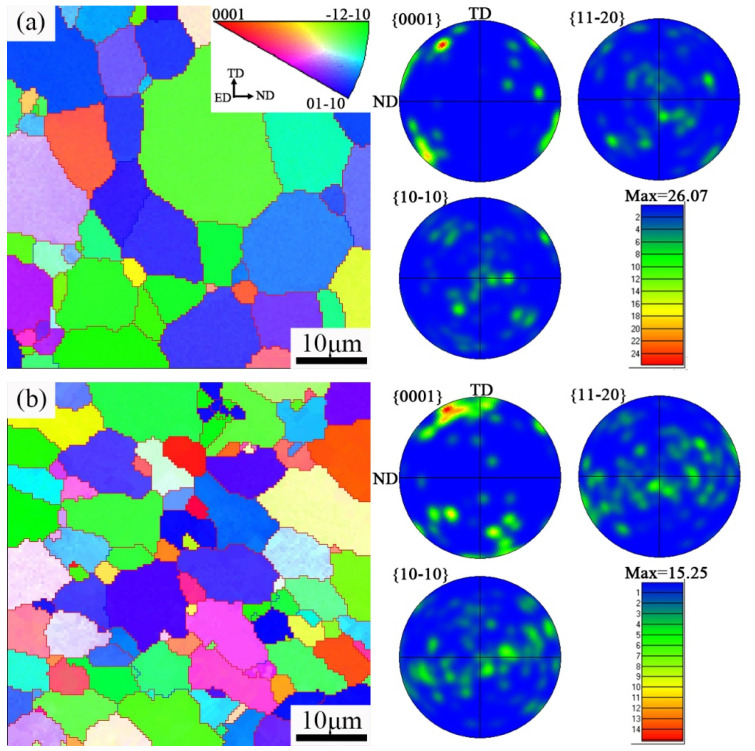
EBSD orientation maps and pole figures of the as-extruded (**a**) EK30 alloy and (**b**) EK30–2.0Ag alloy.

**Figure 5:**
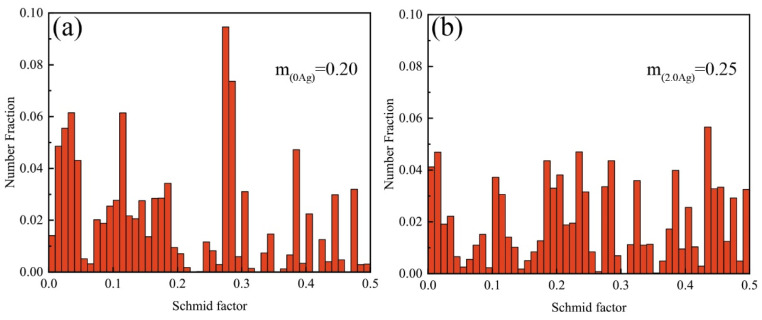
Schmid factor for the {0001} <11$\bar{2}$0> basal slip system of the as-extruded (**a**) EK30 alloy and (**b**) EK30–2.0Ag alloy.

The EBSD orientation maps and pole figures of the as-extruded EK30 and EK30–2.0Ag alloys are shown in Figure 4. The effect of Ag on the grain refinement of the hot extruded alloys can be more clearly observed in the EBSD orientation maps. Furthermore, Ag is also effective at weakening the {0001} basal texture, which can also be attributed to the multi-oriented grains whose formation was induced and promoted during the dynamic recrystallization by the numerous *β*-phase particles [29]. Additionally, the Schmid factors were determined from the basal slip to assess the effect of texture on the mechanical properties. Figure 5 shows the Schmid factors of the {0001} <11$\bar{2}$0> basal slip system for the EK30 and EK30–2.0Ag alloys. The average Schmid factors of the EK30–2.0Ag alloy are slightly higher than those for the EK30 alloy. A higher Schmid factor means that the basal slip is more likely to activate, resulting in a higher elongation of the alloy [30,31].

### 3.3. Tensile Properties

**Figure 6:**
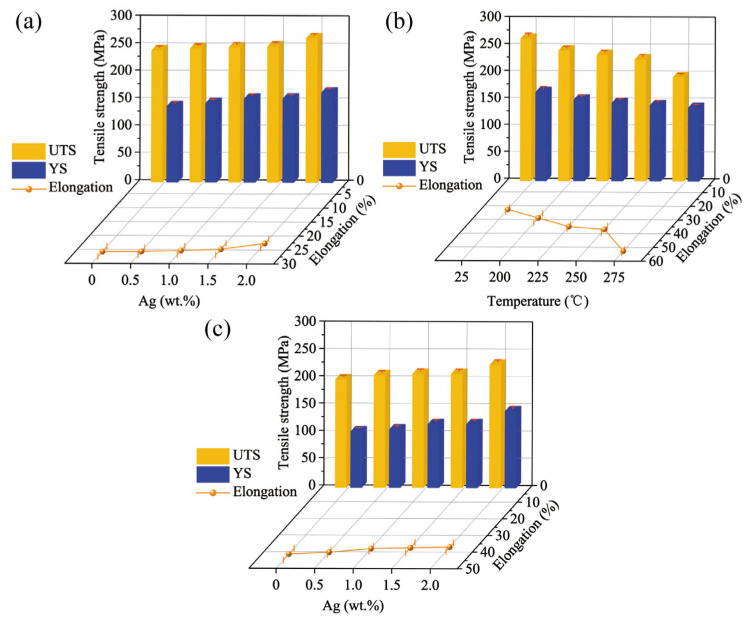
Tensile properties of the as-extruded (**a**) EK30-xAg alloys at 25 °C, (**b**) EK30–2.0Ag alloy at different tensile temperatures, and (**c**) EK30-xAg alloys at 250 °C.

**Figure 7:**
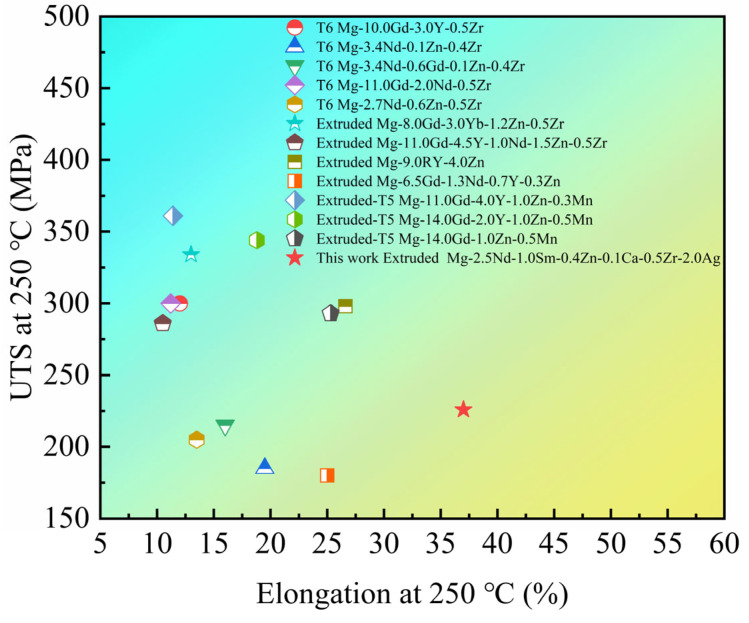
Comparison of tensile properties in different heat-resistant Mg alloys at 250 °C.

Figure 6a shows the ultimate tensile strength (UTS), yield strength (YS), and elongation of the as-extruded EK30-xAg alloys at room temperature. The UTS, YS, and elongation of the EK30 alloy were 240.9 MPa, 139.0 MPa, and 25.8%, respectively. It can be seen that the as-extruded alloys possessed both high strength and ductility. The addition of Ag further enhanced the strength of the as-extruded alloys, which is consistent with the Hall–Petch relationship [32]. The EK30–2.0Ag alloy had comprehensive tensile properties with the UTS, YS, and elongation of 263.7 MPa, 164.8 MPa, and 22.7%, respectively, at room temperature. To examine the high-temperature tensile properties of the as-extruded alloys, tensile tests of the EK30–2.0Ag alloy were conducted at temperatures from 200 °C to 275 °C at 25 °C increments. The tensile properties of the EK30–2.0Ag alloy at high temperatures are shown in Figure 6b. As the tensile temperature increased, the strength of the EK30–2.0Ag alloy decreased, and the elongation increased gradually. Notably, the strength of the EK30–2.0Ag alloy decreased slowly until about 250 °C, and the strength decreased significantly above 250 °C. The UTS of the EK30–2.0Ag alloy dropped from 225.9 MPa at 250 °C to 192.9 MPa at 275 °C. Meanwhile, the elongation of the EK30–2.0Ag alloy increased from 37.0% at 250 °C to 52.7% at 275 °C. The EK30–2.0Ag alloy demonstrated exceptional strength at tensile temperatures up to 250 °C. To compare the strength and elongation of the alloys with different Ag contents at high temperature, the tensile properties were tested at 250 °C, as shown in Figure 6c. The influence of Ag on the high-temperature strength and elongation of the as-extruded EK30-xAg alloys follows the same trend as that at room temperature. The addition of Ag can effectively improve the high-temperature strength of the EK30-xAg alloys. The EK30–2.0Ag alloy exhibited the highest UTS (225.9 MPa) at 250 °C with an increase of 13.8% as compared to the EK30 alloy (198.4 MPa). The addition of Ag slightly reduced the elongation of the alloy at 250 °C; the elongations of the EK30–2.0Ag alloy and the EK30 alloy are 41.5% and 37.0%, respectively. The tensile properties of the as-extruded EK30-xAg alloys at different temperatures are summarized in Table 2. The tensile properties of other heat-resistant Mg alloys at 250 °C are compared in Figure 7 [33,34,35,36,37,38,39,40,41,42]. The research on heat-resistant Mg alloys is mainly focused on Mg-heavy RE alloys. The experimental alloy exhibits lower strength and higher elongation than these Mg-heavy RE alloys. Notably, Mg-heavy RE alloys usually contain large amounts of RE elements. Compared to those, the smaller additions of RE elements in the experimental alloys contributed to their lower density. The combination of good high-temperature strength, excellent elongation, and low density results in the as-extruded EK30-xAg alloy potentially serving as an ideal lightweight material for aerospace applications.

According to the microstructure analysis, adding Ag can effectively refine the *α*-Mg grains and promote the formation of the *β* phase. Thus, the increase in the strength of the as-extruded EK30-xAg at room temperature can be attributed mainly to the grain refinement and *β*-phase strengthening effect of the Ag addition. Additionally, the most significant factor contributing to the strength of the EK30-xAg alloy under high-temperature tensile conditions is the strengthening effect of the *β* phase. In the process of the high-temperature tensile test, the *β*-phase particles located on the *α*-Mg grain boundaries can pin the *α*-Mg grain boundaries [43]. The presence of *β*-phase particles within the *α*-Mg grain can effectively impede dislocation slip. Nevertheless, the coarse size and excessive second phase can also induce stress concentration. The stress concentration around the *β* phase during tensile testing may induce microcrack formation [19,44]. These microcracks may result in a macroscopic fracture during tensile deformation. Thus, the increase in the *β* phase leads to a decrease in the elongation of the Ag-containing alloy. In this regard, the elongation of the alloy gradually decreased with the increase in the Ag content. Notably, the strength of the alloy decreased significantly, while the elongation increased substantially when the tensile temperature was above 250 °C. This is mainly due to more slip systems of *α*-Mg being activated at high temperatures and reduction in the dislocation slip resistance, which increase the elongation of the alloy [45].

### 3.4. Fractography

**Figure 8:**
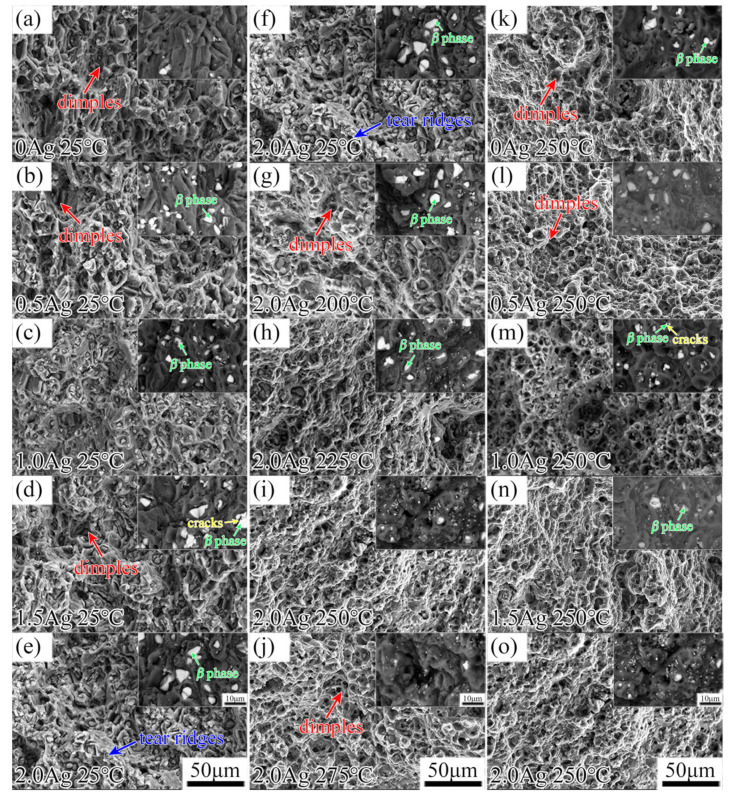
Secondary electron images of the fracture surface of the as–extruded (**a**–**e**) EK30–xAg alloys at 25 °C; (**f**–**j**) EK30–2.0Ag alloy at 25 °C to 275 °C; (**k**–**o**) EK30–xAg alloys at 250 °C. Insets of each subfigure show the corresponding backscattered electron images.

The fracture surface morphologies of the as-extruded EK30-xAg alloys at room temperature and high temperatures are shown in Figure 8. It can be observed that the fracture images of all alloys show numerous dimples and tear ridges. Meanwhile, *β*-phase particles were observed at the bottom of dimples. During tensile deformation, these particles may become the source of cracks. The fracture images of the EK30-xAg alloys showed similar fracture characteristics at the same temperature. As shown in Figure 6 and Table 2, the Ag-containing alloys exhibit excellent elongation close to that of the Ag-free alloys. The fracture mode of the as-extruded EK30-xAg alloys is a typical ductile fracture. In addition, compared to the fracture at 25 °C, more dimples can be observed on the fracture surface at high temperatures, and this characteristic is consistent with the exceptional elongation performance at high temperatures (Figure 6 and Table 2).

## 4. Conclusions

1. The microstructures of as-extruded EK30–xAg alloys consist of *α*–Mg grains and the *β* phase. The addition of Ag increases the *β*-phase content. The *β* phase can promote dynamic recrystallization by inducing a particle-stimulated nucleation mechanism and inhibiting grain growth, which leads to grain refinement and texture weakening.
2. The EK30–2.0Ag alloy exhibits an excellent balance between strength and ductility. Compared to the Ag–free alloy, the ultimate tensile strength of the EK30–2.0Ag alloy increased by 13.8% from 198.4 MPa to 225.9 MPa, while the elongation decreased from 41.5% to 37.0% under the high-temperature tensile conditions at 250 °C. When the tensile temperature increased from 25 °C to 250 °C, the ultimate tensile strength of the EK30–2.0Ag alloy decreased by 14.3%, from 263.7 MPa to 225.9 MPa. The EK30-xAg alloys exhibited ductile fracture behavior at room and high-temperature tensile conditions.
3. This study provided an effective way to achieve the outstanding high-temperature strength and elongation of Mg-light RE alloys via Ag alloying and the IEECAP method.

## Figures and Tables

**Table 1:** EDS results of the points marked in Figure 3 (at.%).

Position	Nd	Sm	Ag	Zn	Ca	Zr	Mg
A	0.27	0.10	0	0.11	0.04	0	99.48
B	5.44	1.13	0	0.99	0.50	0.12	91.82
C	0.25	0.10	0.21	0.11	0.03	0.79	98.51
D	5.01	1.31	1.08	0.91	0.55	0.98	90.16
E	0.20	0.08	0.38	0.09	0.03	0.06	99.16
F	6.08	0.85	5.74	1.37	0.23	0	85.73

**Table 2:** Tensile properties of EK30-xAg alloy at different temperatures.

Alloy	Temperature (°C)	YS (MPa)	UTS (MPa)	Elongation (%)
EK30	25	139.0 ± 3.2	240.9 ± 4.2	25.8 ± 1.8
EK30	250	103.0 ± 2.7	198.4 ± 4.3	41.5 ± 3.6
EK30–0.5Ag	25	144.5 ± 2.8	244.8 ± 4.3	25.7 ± 1.3
EK30–0.5Ag	250	106.8 ± 3.0	206.2 ± 3.4	40.3 ± 1.2
EK30–1.0Ag	25	153.1 ± 4.2	246.5 ± 4.5	25.3 ± 1.5
EK30–1.0Ag	250	116.7 ± 4.3	208.8 ± 2.7	38.0 ± 1.5
EK30–1.5Ag	25	153.4 ± 3.1	248.8 ± 3.2	24.8 ± 2.1
EK30–1.5Ag	250	116.9 ± 3.8	209.0 ± 3.2	37.5 ± 3.1
EK30–2.0Ag	25	164.8 ± 3.6	263.7 ± 5.1	22.7 ± 1.6
EK30–2.0Ag	200	151.1 ± 2.6	242.1 ± 4.1	28.8 ± 3.6
EK30–2.0Ag	225	145.2 ± 3.7	233.9 ± 4.4	35.0 ± 1.4
EK30–2.0Ag	250	140.9 ± 4.5	225.9 ± 3.9	37.0 ± 2.5
EK30–2.0Ag	275	136.3 ± 2.2	192.9 ± 3.7	52.7 ± 1.2

## Data Availability

The data presented in this study are available on request from the corresponding author.
